# Community pharmacists’ cultural competence and awareness in healthcare delivery: a cross-sectional study on perceptions, practices, and demographic influences in the United Arab Emirates

**DOI:** 10.1080/20523211.2025.2552423

**Published:** 2025-09-12

**Authors:** Khalid Awad Al-Kubaisi, Derar H. Abdel-Qader, Karem H. Alzoubi, Abduelmula R. Abduelkarem, Nadia Al Mazrouei, Semira Abdi Beshir, Asim Ahmed Elnour

**Affiliations:** a Department of Pharmacy Practice and Pharmacotherapeutics, College of Pharmacy, University of Sharjah, Sharjah, UAE; b Department of Clinical Pharmacy and Pharmacy Practice, Faculty of Pharmacy and Medical Sciences, The University of Petra, Amman, Jordan; c Department of Pharmacy Practice, Dubai Pharmacy College for Girls, Dubai, UAE; d Clinical Pharmacy Program, College of Pharmacy, Al Ain University, Abu Dhabi, UAE; e Research Institute of Medical & Health Sciences (RIMHS), University of Sharjah, Sharjah, UAE

**Keywords:** Cultural competence, community pharmacists, cross-sectional study, healthcare delivery, perceptions and practices

## Abstract

**Background:**

Community pharmacists (CPs) are crucial in the healthcare system, particularly in providing culturally sensitive care to diverse populations.

**Method:**

This cross-sectional study assessed the cultural competence and culturally competent behaviours of 360 licensed CPs practicing in Dubai, Sharjah, and Ajman, focusing on the influence of demographic characteristics, training, and workplace support.

**Result:**

Most participants were aged 31-40, mostly non-Arab and bilingual. Although 88.9% had lived abroad for over three years, only 24.4% viewed themselves as culturally competent. The mean cultural awareness score was 44.69, indicating moderate to high awareness. An independent samples t-test revealed that CPs who had received cultural diversity training scored significantly higher on the cultural awareness scale (M = 46.17, SD = 9.84) than those without training (M = 43.82, SD = 10.29), t(358) = 2.121, *p* = .035. A statistically significant association was found between previous cultural diversity training and self-perceived competence (χ²(4) = 19.933, *p* < .001). Furthermore, a strong association was observed between perceived adequacy of staffing and workflow and self-perceived competence (χ²(8) = 37.523, *p* < .001; Cramér's V = 0.228). Additionally, one-way ANOVA tests showed no significant differences in cultural competence behaviour scores observed across demographic and workplace variables.

**Conclusion:**

This study highlights the need for cultural competence training for CPs to improve patient-centred care in diverse healthcare environments in the United Arab Emirates.

## Background

Cultural competency can be defined as ‘the dynamic process of acquiring the ability to provide effective, safe, and quality care to the patients by considering their different cultural aspects’(Cai, 2016). This concept has increasingly become a cornerstone in the provision of patient-centred care, particularly in the context of globalisation and migration, which have led to more culturally diverse patient populations. It can improve medication adherence among patients of various ethnic origins (Ervin et al., 2006), increase patient satisfaction, and help combat prejudice in the healthcare system (Antón-Solanas et al., 2021), and is thus essential for healthcare providers (Corsi et al., 2018), such as community pharmacists, primarily in multicultural environments such as the United Arab Emirates (UAE). For example, during Ramadan, Muslims usually refrain from consuming anything by mouth between dawn and sunset, and their medication schedules may need to be adjusted. Consequently, the cultural competence of community pharmacists is vital (Atif et al., 2020; Farmer et al., 2019; Gaither, 2021; Rayes et al., 2015).

Cultural competence in healthcare is not only about understanding and respecting cultural differences but also about adapting healthcare services to meet the cultural needs of patients. This adaptation can lead to better health outcomes, increased patient trust, and more effective communication between healthcare providers and patients (Betancourt et al., 2016). This is even more fundamental in culturally diverse environments such as the UAE, home to more than 200 nationalities (Haq & Medhekar, 2020a; 2020b; Shukla & Kulshreshtha, 2020). The country also attracted almost half a million medical tourists in 2021 from Arab, and Middle East, Asian countries, the United States, the European Union, and other regions (Ahmed et al., 2024; Al-Talabani et al., 2019; Haq & Medhekar, 2020a; 2020b; Heydari et al., 2019; Hosseini & Mirzaei, 2021; Shukla & Kulshreshtha, 2020). The influx of medical tourists further underscores the necessity for culturally competent care, as these patients bring with them diverse health beliefs, practices, and expectations (Henderson et al., 2018). In addition, most community pharmacists working in the UAE are mainly expatriates from many countries, with only a few Emiratis (Haq & Medhekar, 2020b; Heydari et al., 2019; Shukla & Kulshreshtha, 2020). Therefore, to deliver culturally competent care, community pharmacists must overcome additional barriers to communication and cultural understanding from patients of different languages and cultural backgrounds (Atif et al., 2020). These barriers can impede the delivery of effective healthcare services and can lead to misunderstandings, misdiagnoses, and patient dissatisfaction (Dushenkov et al., 2020a). The situation in the Emirates is even more challenging because pharmacists must collaborate with staff members from various cultural and language backgrounds to ensure the delivery of culturally competent care.

However, most studies on the cultural competency of healthcare providers have been carried out in Western countries (Chen et al., 2021; Dushenkov et al., 2020b; Henderson et al., 2018; Karakuş et al., 2021; Mohammad et al., 2021a; Nkhoma et al., 2022; Osmancevic et al., 2023). These studies have provided valuable insights into the importance of cultural competence in healthcare, but they may not fully apply to the unique cultural context of the UAE and the broader Middle East region. These studies have also focused on nurses, pharmacy students, and pharmacy educators rather than community pharmacists (Antón-Solanas et al., 2021; Chen et al., 2021; de las Mercedes Martínez Sánchez & Warrent Salmon, 2022; Vázquez-Sánchez et al., 2023; Wang et al., 2018) Community pharmacists play a crucial role in the healthcare system, often serving as patients’ first point of contact. Their interactions with patients can significantly influence health outcomes, making it essential to assess and enhance their cultural competence (Schwarz et al., 2015a).

To the best of the researcher’s knowledge, no published study investigated the level of cultural competency among pharmacists working in the GCC or the Middle East. This gap in the literature highlights the need for research that specifically addresses the cultural competence of community pharmacists in these regions. Understanding the current level of cultural competence and the factors that influence it can provide valuable insights for developing targeted interventions and training programmes*.* This study aimed to assess the cultural competence of community pharmacists in the UAE and identify influencing factors using the Cultural Competence Assessment Scale CCA (Harris-Haywood et al., 2014; Schwarz et al., 2015b).

By conducting this study, we aim to provide a comprehensive assessment of cultural competence among community pharmacists in the UAE, identifying the levels of cultural awareness, culturally competent behaviours, and the factors influencing self-perceived competence using a validated assessment tool. This research addresses a critical gap in the regional literature by systematically evaluating how structured cultural training and workplace support impact pharmacists’ competence. The findings have significant implications for healthcare education and policy in the Gulf region, emphasising the urgent need for integrating structured cultural competence and cultural humility training into pharmacy curricula, continuing professional development (CPD) programmes, and licensure requirements. Furthermore, this study advocates organisational changes that enhance workplace support systems to foster culturally sensitive pharmaceutical care, thereby improving patient-centred services for the UAE’s diverse population.

## Materials and methods

### Study design and population

This study used a cross-sectional study design was undertaken between August and October 2023. The main advantages of this type of study are the ease of administration (as a self-administered survey), the measurement of multiple outcomes simultaneously, the relatively large sample it permits for the analysis, and the provision of valuable information in planning health services. To ensure rigour, we pre-tested the survey instrument with a small sample (n = 10) of community pharmacists to identify any ambiguities or issues with question interpretation. Feedback from this pilot was used to refine the questionnaire. Additionally, we conducted a reliability analysis to ensure internal consistency of the scales used (Cronbach’s alpha > 0.7).

### Study population and sampling

According to the Dubai Health Authority (DHA), there were 4000 pharmacists in the UAE in 2019. Calculating the sample size needed was based on a confidence level of 95% and a confidence interval of 5; therefore, the sample size required for this study was 351 participants. We used a cluster sampling technique in which subgroups of the community pharmacy population were used as the sampling unit. The sampling frame was the address list of all registered community pharmacies in the Yellow Pages directory. Therefore, the names of pharmacies in the three main emirates of UAE (Dubai, Sharjah, and Ajman) were entered into an Excel spreadsheet, each assigned a number. Accordingly, four hundred pharmacies were randomly chosen using software to generate random numbers, assuming each pharmacy has one community pharmacist.

To enhance the rigour of our sampling process, we employed a stratified random sampling method to ensure representation across different emirates and types of pharmacies (chain vs. independent). This approach helps reduce sampling bias and increase our findings’ generalisability (Levy & Lemeshow, 2020).

This study investigated the cultural competency among only licensed community pharmacists working in community pharmacies. Hospital pharmacies, pharmacy students, other healthcare providers, and administrative staff were excluded from the study.

The research team contacted the pharmacists at their pharmacies during regular business hours. They identified themselves as independent researchers unaffiliated with the Ministry of Health (MOH), Dubai Health Authority (DHA), or any other health regulatory body. They assured the pharmacists that their responses would remain confidential and only be used for the study. The team explained their research goals and requested the pharmacists’ voluntary participation in the survey. They also provided contact information in case the pharmacists had any questions or concerns about the study. Additionally, participants were assured that their involvement would have no adverse impact on their professional standing, as their responses would remain anonymous and confidential and would not be shared with any third party. To further ensure rigour, we implemented a standardised script for all researchers to follow when contacting participants, ensuring consistency in the information provided and reducing potential researcher bias. We also logged all contacts and responses to monitor response rates and follow up on non-respondents (Dillman et al., 2014). The team emphasised the importance of candid feedback from the pharmacists to ensure the validity and reliability of the study results. The researchers aimed to create a collaborative and supportive environment for pharmacists to share their experiences and perspectives openly.

## Data collection-questionnaire

### Research instruments

The questionnaire was disseminated in English via Google Forms and structured into five sections. These sections included sociodemographic inquiries and four sub-scales: self-perceived cultural competence, cultural awareness and sensitivity (CAS), culturally competent behaviour (CCB), and attitudes towards cultural training, all of which were adapted from the Cultural Competence Assessment (CCA) instrument (Doorenbos et al., 2005). To establish the validity of our adapted instrument, we conducted a confirmatory factor analysis (CFA) to ensure that the factor structure of the CCA instrument held true in our sample. This step is crucial for validating the use of adapted instruments in different cultural contexts (Brown, 2015). The self-reported CCA has been translated into various languages and then psychometrically assessed in each language (Italian, Spanish, Korean, and Slovakian). It has been demonstrated to be a reliable and valid instrument for assessing cultural competency in healthcare (Chae et al., 2018; Vázquez-Sánchez et al., 2023).

The first section encompassed the sociodemographic profile of participants, encompassing variables such as age, gender, ethnicity, level of English proficiency, and multilingual capabilities. Additionally, participants provided details regarding their educational background, years of professional experience, and employment setting, distinguishing between chain and independent pharmacies. Further characteristics included the location of the pharmacy, categorised as standalone, within a shopping centre, or adjacent to a medical centre, as well as the nature of customer interaction (regular, pass-by, or both). Moreover, participants indicated the availability of consultation areas within their workplace, ranging from no designated area to semi-private or fully private spaces. Information regarding weekly working hours, the volume of daily patient interactions, and the adequacy of workflow, staffing, and time management were also captured. Lastly, participants disclosed whether they had resided abroad for over three years, providing contextual insight into their exposure to diverse cultural environments.

The second section focused on participants’ self-perceived cultural competence, wherein individuals assessed their level of cultural proficiency using a 5-point Likert scale. This scale ranged from descriptors such as ‘very incompetent’ and ‘somewhat incompetent’ to ‘neither competent nor incompetent,’ ‘somewhat competent,’ and ‘very competent.’

The third section comprised the CAS sub-scale, encompassing questions 1–12, rated on a 5-point Likert scale from ‘not at all’ to ‘very often.’ The subsequent fourth section encompassed the CCB subscale, which included questions 1–14, also rated on a 5-point Likert scale from ‘not at all’ to ‘very often.’ Elevated scores on both the CAS and CCB subscales signify a heightened perception of cultural competence.

The final section of the survey inquired about participants’ attitudes regarding the significance of cultural diversity and/or multicultural healthcare training for health professionals. Responses were elicited using a 5-point Likert scale, ranging from ‘not at all’ to ‘very often’ (Appendix).

### Data analysis

Data was analysed using the statistical software program IBM SPSS Statistics 27. Descriptive statistics were used to summarise participant demographics, while chi-square tests (with Cramér’s V) assessed associations between categorical variables such as previous training and self-perceived competence. Independent samples t-tests (with Cohen’s d) and one-way ANOVA (with eta squared) compared mean cultural awareness and culturally competent behaviour scores across demographic and workplace variables, with Levene’s test confirming the homogeneity of variances. Statistical significance was determined to be *P* < 0.05. To ensure the rigour of our data analysis, we conducted a power analysis to confirm that our sample size was adequate to detect meaningful effects. Additionally, we used multiple imputation techniques to handle any missing data, ensuring that our results were not biased by missing responses (Enders, 2022). We also employed multivariate analysis to control for potential confounding variables and to explore interactions between different demographic and workplace variables.

## Results

### Demographic characteristics

The study had a high participation rate, with 360 of the 400 targeted pharmacies agreeing to participate (86.5%). The average participant age was 31.7 years (SD = 4.6), with more than half (55%) falling within the 31–40 age group. The gender distribution was relatively balanced, with 188 males (52.2%) and 172 females (47.8%). Two-thirds (65.3%) of participants were non-Arab. Nearly all pharmacists spoke two languages (98.9%), and most (88.9%) lived abroad for over three years. About 45.8% reported fluent English proficiency.

Most participants (75.8%) had fewer than 11 years of work experience, and 92.5% held a bachelor's degree as their highest educational qualification. Most pharmacists (68.1%) worked in chain pharmacies, and 76.9% were employed in standalone locations. Over half (54.7%) interacted with regular and pass-by customers, while 88.3% reported serving two patients simultaneously. Additionally, 50.8% saw more than 30 patients daily, and 61.4% had access to a semi-private consultation area. Detailed sociodemographic characteristics are presented in Table 1.

**Table 1.** Demographic distribution characteristics of the participants (N = 360).

Characteristic	Frequency (%)
*Age (years)*	
Mean (sd)	
*Gender*	
Female	172 (47.8)
Male	188 (52.2)
*Nationality*	
Arab	125 (34.7)
Non-Arab	235 (65.3)
*English Level*	
*Basic*	46(12.8)
*Conversational*	109(30.3)
*Fluent*	165(45.8)
*Proficient*	40(11.1)
*Speak a Second Language*	
*Yes*	356(98.9)
*No*	4(1.1)
*Type of pharmacy*	
Chain pharmacy	245 (68.1)
Independent pharmacy	115(31.9)
*Working hours/week*	
1–8	8(4)
> 8	192(96)
*Lived abroad for more than three years*	
Yes	320(88.9)
No	40(11.1)
*Work experience (years)*	
*1-10*	273 (75.8)
*11-20*	74(20.6)
*>20*	13(3.6)
*Number of patients per day*	
*<20*	40(11.9)
*20-30*	143(37.2)
*>30*	183(50.8)
*Type of customer*	
*Regular*	58(16.1)
*Pass by*	105(29.2)
*Both*	197(54.7)
*Interact with two patients simultaneously.*	
*Yes*	318(88.3)
*No*	42(11.7)
*Place of the pharmacy*	
*Standalone*	277(76.9)
*Shopping center*	44(12.2)
*Next to the Medical Center*	39(10.8)
*Consultation area*	
*No*	96(26.7)
*Semiprivate area*	221(61.4)
*Private consultation*	43(11.9)
*Adequate workflow, time, and staff*	
*No staff/No time*	16(44)
*Yes, workflow/not enough staff*	148(41.1)
*Yes, workflow/time/staff*	195(54.2)
*Previous training in cultural diversity*	
*Yes*	132(36.7)
*No*	228(63.3
*Self-perceived cultural competence*	
*Very incompetent*	17(4.7)
*Somewhat incompetent*	22(6.1)
*Neither competent nor incompetent*	88(24.4)
*Somewhat competent*	22(6.2)
*Very competent*	177(49.2)
*Educational Qualification*	
Bachelor of Pharmacy	333(92.5)
Diploma	13(3.6)
Master (M.S.)	14(3.9)

### Attitude towards cultural competency training

**Figure 1.**
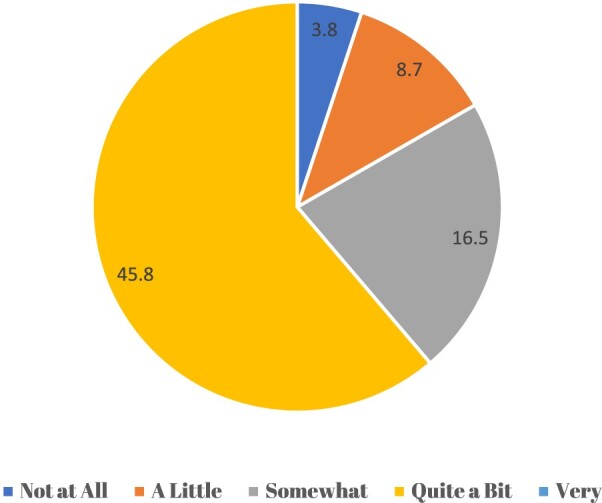
Attitude toward cultural competence training (N=360).

The majority of pharmacists viewed cultural competency training as important. While only 3.9% felt it was ‘Not at All’ important, a significant proportion considered it either ‘Quite a Bit’ (46.4%) or ‘Very’ important (25.8%). These results reflect a strong recognition among participants of the value of training in cultural diversity (Figure 1).

### Perceived cultural competence

**Figure 2.**
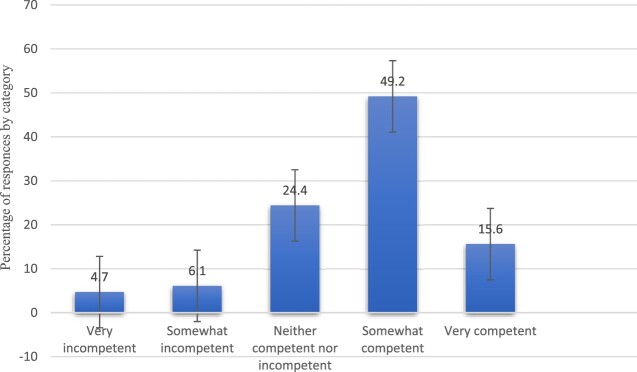
Self-perceived cultural competence of pharmacists (N=360).

Figure 2 shows that approximately 24.4% of respondents perceived themselves as either culturally competent or incompetent. Specifically, 6.6% reported feeling somewhat culturally incompetent, while 4.7% described themselves as very culturally incompetent. In contrast, 48.4% felt somewhat culturally competent, and 15.6% described themselves as very culturally competent. Notably, 63.3% of participants reported no previous training in cultural competency.

### Association between training and perceived competence

A chi-square test revealed a statistically significant association between previous cultural diversity training and self-perceived competence (χ²(4) = 19.933, *p* < .001), with a small to moderate effect size (Cramér's V = 0.235). Similarly, a strong association was observed between perceived adequacy of staffing and workflow and self-perceived competence (χ²(8) = 37.523, *p* < .001; Cramér's V = 0.228), suggesting the influence of workplace support on pharmacists’ confidence in their cultural competence.

No significant associations were found between self-perceived competence and other variables, including race (χ²(4) = 9.274, *p* = 0.055), gender (χ²(4) = 1.369, *p* = 0.850), living abroad (χ²(4) = 4.489, *p* = 0.344), or serving two patients simultaneously (χ²(4) = 4.737, *p* = 0.315) as shown in table 2.

**Table 2.** Chi-Square test results for association between training and self-perceived competence.

"Variable"	χ² (df)	*p*-value
Previous Training	19.933 (4)	< .001
Race	9.274 (4)	0.055
Workload Adequacy	37.523 (8)	< .001
Gender	1.369 (4)"	0.850
Lived Abroad	4.489 (4)	0.344
Serve two patients	4.737 (4)	0.315

### Cultural awareness and sensitivity

The mean cultural awareness score was 44.69 (SD = 10.18), indicating a moderate to high level of awareness overall. Scores ranged from 14 to 70, with the majority clustered around the midpoint of the scale. The highest scoring items were Q6: ‘I enjoy working with people who are culturally different from me’ (M = 3.97, SD = 1.03) and Q2: ‘I believe everyone should be treated with respect regardless of their cultural heritage’ (M = 3.78, SD = 1.34). Items with the lowest scores included Q7: ‘Race is the most important factor in determining a person’s culture’ (M = 2.68, SD = 1.29) and Q11: ‘If I know about a person’s culture, I don’t need to assess their personal preferences for health services’ (M = 2.72, SD = 1.33). These results suggest that pharmacists strongly support respect and openness to cultural diversity while rejecting simplistic assumptions about cultural identity (Table 3).

**Table 3.** Pharmacists’ responses to the Cultural Awareness and Sensitivity (CAS) assessment (N = 360).

Items	Not at All n (%)	A Little n (%)	Somewhat n (%)	Quite a Bit n (%)	Very often n (%)	Mean (SD)
1. Individual people may identify with more than one cultural group.	57(15.8)	61(16.9)	106(29.4)	87(24.2)	49(13.6)	3.03(1.26)
2.I believe everyone should be treated with respect regardless of their cultural heritage.	28(7.8)	46(12.8)	54(15)	73(20.3)	157(43.6)	3.78(1.34)
3. Language barriers are the only difficulties for recent expats to the UAE.	72(20)	85(23.6)	83(23.1)	85(23.6)	35(9.7)	2.79(1.27)
4. I understand that people from different cultures may define the concept of ‘health care’ in different ways.	30(8.3)	76(21.1)	94(26.1)	82(22.8)	78(21.7)	3.28(1.25)
5. I think that knowing about different cultural groups helps direct my work with individuals, families, groups, and organisations	30(8.3)	64(17.8)	88(24.4)	92(25.6)	86 (23.9)	3.39(1.25)
6. I enjoy working with people who are culturally different from me.	6(1.7)	30(8.3)	71(19.7)	116(32.2)	137(38.1)	3.97(1.031)
7. Race is the most important factor in determining a person’s culture.	86(23.9)	82(22.8)	90(25.0)	66(18.3)	36(10.0)	2.68 (1.29)
8. People with a common cultural background think and act alike.	44(12.2)	63(17.5)	100(27.8)	104(28.9)	49(13.6)	3.14(1.21)
9. Many aspects of culture can influence health and healthcare.	30(8.3)	63(17.5)	85(23.6)	124(34.4)	58(16.1)	3.33(1.18)
10. Aspects of cultural diversity need to be assessed for each individual, group, and organisation’s Culturally competent behaviour	30(8.3)	53(14.7)	116(32.2)	109(30.3)	52(14.4)	3.28(1.135)
11. If I know about a person’s culture, I don’t need to assess their personal preferences for health services.	101(28.1)	52(14.4)	82(22.8)	97(26.9)	28(7.8)	2.72(1.33)
12. Spiritual and religious beliefs are important aspects of many cultural groups.	44(12.2)	52(14.4)	94(26.1)	126(35.0)	44(12.2)	3.21(1.19)
13. Individual people may identify with more than one cultural group	47(13.1)	52(14.4)	104(28.9)	120(33.3)	37(10.3)	3.13(1.18)
14. Language barriers are the only difficulties for recent expats in the UAE.	48(13.3)	84(23.3)	95(26.4)	99(27.5)	34(9.4)	2.96(1.93)

### Differences in cultural awareness by demographics

An independent samples t-test revealed that pharmacists who had received cultural diversity training scored significantly higher on the cultural awareness scale (M = 46.17, SD = 9.84) than those without training (M = 43.82, SD = 10.29), t(358) = 2.121, *p* = .035. The effect size was small (Cohen's d = 0.232). No significant differences were found based on gender (t(358) = 1.526, *p* = .128), nationality (t(358) = −0.802, *p* = .423), or previous international experience (t(358) = −0.157, *p* = .875) as displayed in table 4.

**Table 4.** Independent samples t-test results comparing cultural awareness scores by demographic variables (N = 360).

Comparison	Mean (SD) – Group 1	Mean (SD) – Group 2	Levene’s Test (F, *p*)	t(df), *p*-value	95% CI	Cohen’s d (Effect Size)
Gender (Female vs Male)	45.54 (9.79) – Female	43.90 (10.49) – Male	1.621, *p* = .204	"t(358) = 1.526, *p* = .128	[−0.47, 3.74]	0.161 (Small)
Race (Arab vs non Arab)	Group 1 (Arab), 44.10, (9.35)	Group 2 (Non-Arab)45.00 (10.60)	F = 2.765, *p* = .097	t(358) = −0.802, *p* = .423	[−3.12, 1.31]	−0.089
Training in Cultural Diversity (Yes vs No)	46.17 (9.84) – Trained	43.82 (10.29) – Not Trained	1.265, *p* = .262	t(358) = 2.121, *p* = .035	[0.17, 4.53]	0.232 (Small)
Living Abroad (Yes vs No)	44.66 (10.18) – Lived Abroad	44.92 (10.27) – Not Lived Abroad	0.030, *p* = .862"	t(358) = −0.157, *p* = .875	[−3.63, 3.09]	−0.026 (Negligible)
Chain/Banner Group Pharmacy vs Independent Pharmacy	44.32 (10.44) – Chain/Banner Group Pharmacy	45.46 (9.59) – Independent Pharmacy"	2.073, *p* = .151"	t(358) = −0.989, *p* = .323	[−3.40, 1.12]	−0.112 (Negligible)

### Culturally competent behaviour (CCB)

The mean score for culturally competent behaviour was 49.23 (SD = 8.38) out of 70, indicating a moderate overall level. Item 11 (‘I welcome feedback from clients about how I relate to people from different cultures’) scored the highest (M = 4.06, SD = 0.90). In contrast, items related to including cultural assessments and using resource materials (Items 1 and 3) scored lowest (M = 3.15). Table 5 summarises the mean scores of culturally competent behaviour (CCB) items among CPs.

**Table 5.** Mean scores of Culturally Competent Behaviour (CCB) items (N = 360).

Items	Not at All n (%)	A Little n (%)	Somewhat n (%)	Quite a Bit n (%)	Very n (%)	Mean (SD)
1. I include cultural assessments when I do individual or organisational evaluations.	35(9.7)	49(13.6)	127(35.3)	124(34.4)	25(6.9)	3.15(1.06)
2. When identifying new people in my work or pharmacy, I seek information on cultural needs.	40(11.1)	58(16.1)	91(25.3)	143(39.7)	28(7.8)	3.17(1.13)
3. Resource books and other materials are available to help me learn about people from different cultures.	41(11.4)	54(15.0)	103(28.6)	133(36.9)	29(8.1)	3.15(1.13)
4. I use a variety of sources to learn about other people’s cultural heritage.	31(8.6)	58(16.1)	101(28.1)	139(38.6)	31(8.6)	3.23(1.09)
5. I ask people to tell me their own explanations of health and illness.	15(4.2)	36(10.0)	104(28.9)	144(40.0)	61(16.9)	3.56(1.01)
6. I ask people to tell me about their expectations of health services.	15(4.2)	37(10.3)	99(27.5)	142(39.4)	67(18.6)	3.58(1.03)
7. I avoid using generalisations to stereotype groups of people.	14(3.9)	34(9.4)	79(21.9)	134(37.2)	99(27.5)	3.75(1.07)
8. I recognise potential barriers to service that different people might encounter	13(3.6)	25(6.9)	105(29.2)	155(43.1)	62(17.2)	3.63(0.96)
9. I remove obstacles for people of different cultures when identifying service barriers.	5(1.4)	21(5.8)	102(28.3)	152(42.2)	80(22.2)	3.78(0.90)
10. I remove obstacles for people of different cultures when people identify barriers to me.	6(1.7)	28(7.8)	92(25.6)	150(41.7)	84(23.3)	3.77(0.94)
11. I welcome feedback from clients about how I relate to people from different cultures.	4(1.1)	15(4.2)	69(19.2)	139(38.6)	133(36.9)	4.06(0.909)
12. I find ways to adapt my services to individual and group cultural preferences.	8(2.2)	21(5.8)	78(21.7)	152(42.2)	101(28.1)	3.88(0.959)
13. I document cultural assessments if I provide direct client services.	43(11.9)	37(10.3)	110(30.6)	132(36.7)	38(10.6)	3.24(1.18)
14. I document the adaptations I make with clients if I provide direct client services.	41(11.4)	47(13.1)	104(28.9)	120(33.3)	48(13.3)	3.24(1.18)

### One-Way anova: factors influencing CCB scores

One-way ANOVA tests showed no significant differences in CCB scores across various demographic and workplace variables. For age, the results were F(2, 357) = 0.760, *p* = 0.480, η² = 0.004. For gender, F(1, 358) = 0.090, *p* = 0.728, η² = 0.000. Nationality showed F(1, 358) = 3.181, *p* = 0.075, η² = 0.009. English proficiency yielded F(3, 356) = 0.192, *p* = 0.902, η² = 0.002, and second language ability showed F(1, 358) = 0.453, *p* = 0.501, η² = 0.001. For type of pharmacy, the test was F(1, 358) = 0.203, *p* = 0.653, η² = 0.001. Weekly working hours produced F(3, 356) = 2.167, *p* = 0.092, η² = 0.018. Educational qualification had F(2, 357) = 0.832, *p* = 0.436, η² = 0.005. Consultation area yielded F(2, 357) = 0.046, *p* = 0.955, η² = 0.000, and workflow adequacy was F(2, 357) = 0.367, *p* = 0.693, η² = 0.002. These findings suggest that demographic or workplace characteristics do not significantly influence culturally competent behaviour, underscoring the need for broader strategies to support and enhance cultural competence in pharmacy practice (Table 6).

**Table 6.** One-way ANOVA results for demographic and workplace variables influencing CCB scores (N = 360).

Characteristic	CCB*Mean (SD)	*F*	*η2*	P-value **
*Age (years) 20–30 31–40 >41*	49.326 (8.26) 49.36 (8.96) 46.46(7.57)	F(2, 357) = 0.760	0.004	0.480
*Gender* Female Male	49.06(8.18) 49.37(8.58)	F(1, 358) = 0.090	0.000	0.728
*Nationality* Arab Non-Arab *English Level Basic Conversational Fluent Proficient Speak a Second Language Yes No*	50.34(7.65) 48.63(8.70) 47.58 49.73 49.28 49.52 49.25 46.75	F(1, 358) = 3.181 F(3, 356) = 0.192, F(1, 358) = 0.453	0.009 0.002 0.001	0.075 0.902 0.501
*Type of pharmacy* Chain pharmacy Independent pharmacy	49.51 48.63	F(1, 358) = 0.203	0.001	0.653
*Working hours/week 1–16 17–31 32–40 >40 h Lived abroad for more than three years* Yes No *Number of patients per day <20 20–30 >30*	49.33 49.30 49.18 49.17 49.37 48.07 50.46(5.81) 49.65(9.09) 48.62(8.33)	F(3, 356) = 2.167 F(1, 358) = 0.490 F(2, 357) = 0.543	0.018 0.001 0.003	0.092 0.485. 0.582

*5-likert Scale: very (5) – Quite a Bit – Somewhat – A Little – Not at All (0).

**Significant *P* < 0.05.

***There was homogeneity of variances for scores, the *p*-value for Levene's Test was >0. 05.

**Significant *P* < 0.05.

***There was homogeneity of variances for scores, the *p*-value for Levene's Test was >0. 05.

## Discussion

This study highlights important gaps and opportunities in cultural competence among community pharmacists in the Emirates. While pharmacists reported moderate levels of cultural awareness and culturally competent behaviour, only those with previous cultural diversity training demonstrated significantly higher awareness scores, underscoring the value of structured education. Most demographic factors – including age, gender, nationality, English proficiency, and work setting – did not significantly influence cultural competence levels. However, strong associations were found between pharmacists’ self-perceived competence and both previous training (*p* < .001) and adequate staffing/workflow conditions (*p* < .001). These findings suggest that improving cultural competence in pharmacy practice requires more than just diverse staffing it demands investment in targeted training and supportive work environments. The study provides actionable insights for healthcare policymakers and educators to design culturally focused professional development programs and workplace improvements that support equitable and sensitive patient care.

These results are consistent with a study conducted in Canada, which found that pharmacists who participated in cultural competence training programmes were better equipped to meet the needs of Indigenous populations, leading to improved health outcomes and patient satisfaction (Smith et al., 2022). Additionally, research in New Zealand highlighted the importance of cultural safety training for pharmacists, which not only enhanced their cultural awareness but also improved their ability to provide patient-centred care to Māori communities (Johnson et al., 2023).

Our findings showed that community pharmacists in the Emirates are primarily young, bilingual, and possess international experience. Nevertheless, despite this advantageous demographic, over 60% of participants reported no previous training in cultural competence, a deficiency also noted in studies from the UK, Australia, and the US (McCann et al., 2023; Mohammad et al., 2021b; Movafagh & Adams, 2023). For example, a study carried out in the United States, which focused on the healthcare system's engagement with patients of Asian descent, highlights that community pharmacists perceived themselves as inadequately equipped to address the needs of this demographic resulting in poorer patient engagement, leading to diminished patient engagement, inconsistent treatment outcomes, and an aggravation of health disparities among ethnic minority groups when compared to their White counterparts (Movafagh & Adams, 2023). Furthermore, many pharmacists lacked access to private consultation areas, which may limit opportunities for culturally sensitive interactions, echoing challenges in similar pharmacy settings in Kuwait and Saudi Arabia (Alnesef et al., 2024; Alrasheed et al., 2024; Hatting et al., 2016). This aligns with findings from a study in Sweden, where pharmacists reported similar challenges in providing culturally sensitive care due to a lack of private consultation spaces and structured training programmes (Andersson et al., 2022). The study emphasised the need for infrastructure enhancements and formalised training to improve cultural competence among pharmacists. In total, these findings underscore the importance of formalised training and infrastructure enhancements, aligning with global recommendations to integrate cultural competence into pharmacy education and standards (Gibson & White, 2019; Jarrar et al., 2023; Wheeler et al., 2022)

A key finding of this study was the strong positive attitude among pharmacists towards cultural competency training, with over 70% recognising it as important to their professional role. Despite this, nearly two-thirds had never received formal training in this area, indicating a substantial gap between perceived importance and actual educational exposure. The disparity between pharmacists’ perceived importance of cultural competency training and their actual educational exposure can be attributed to several interrelated factors. These include variations in curriculum implementation, demographic influences, and the conceptualisation of cultural competence itself (Britton et al., 2016; Echeverri & Dise, 2017; Mohammed et al., 2024; Okoro et al., 2012; Okoro et al., 2015; Robinson-Barella et al., 2023). This gap is further supported by a study in Germany, which found that while pharmacy students recognised the value of cultural competency training, they often did not receive adequate exposure during their education (Müller et al., 2023). The study recommended integrating cultural competence training into the pharmacy curriculum to better prepare students for diverse patient populations. For example, a study found that while pharmacy students recognise the value of cultural competency and humility in their education, they seek more structured exposure and curriculum integration to build confidence and envision themselves as culturally competent pharmacists, leading to actionable recommendations for enhancing pharmacy education in alignment with UK standards (Robinson-Barella et al., 2023).

Moreover, our study found a statistically significant association between previous cultural training and pharmacists’ self-perceived competence, as well as between workplace support (adequate staffing and workflow) and perceived competence. These results reinforce evidence from earlier work, showing that an inclusive workplace culture and supportive office management systems – particularly those offering cross-cultural training – are critical for enhancing employees’ Cultural Intelligence (Ifran & Dhowi, 2024). Similar findings were reported in a study conducted in Japan, where pharmacists who received cultural competence training and worked in supportive environments demonstrated higher levels of cultural intelligence and patient satisfaction (Tanaka et al., 2022). The study highlighted the importance of ongoing training and supportive workplace policies in enhancing cultural competence. Therefore, we recommend that health authorities, such as the Ministry of Health and Prevention (MOHAP), SEHA, and EHS, mandate culturally responsive care training as part of licensure renewal and onboarding programmes for pharmacists. Furthermore, pharmacy managers across community settings should ensure adequate staffing and well-organised workflows to reduce stress and free up pharmacists’ time for meaningful patient interaction.

The lack of significant associations between self-perceived competence and demographic factors, such as gender, nationality, or international experience, further suggests that lived experience alone is insufficient for developing cultural skills without structured support. This finding aligns with previous research indicating that, although these factors may influence initial exposure to cultural diversity, they do not replace the need for structured training and education to develop true competence (Rodgers & Furcron, 2019). This is supported by a study in South Africa, which found that while demographic diversity among healthcare providers can enhance cultural exposure, it does not necessarily translate to improved cultural competence without structured training and supportive policies (Nkosi et al., 2023). The study emphasised the need for ongoing education and training to develop cultural competence among healthcare providers.

Pharmacists in this study demonstrated moderate to high levels of cultural awareness and culturally competent behaviour, with top-rated responses reflecting inclusive values such as respect for cultural differences, openness to feedback from clients of diverse backgrounds, and enjoyment in working with multicultural populations. The highest scoring items reflect foundational principles of patient-centred care, emphasising individual dignity and openness. In contrast, lower scores on items like equating race with culture or generalising cultural knowledge indicate a rejection of cultural stereotyping. This supports the idea that pharmacists in this study are not merely acquiring cultural knowledge but are embracing an ongoing, reflective approach that acknowledges power dynamics and the individuality of each patient, consistent with the principles of cultural humility that enhance patient-provider communication and care quality. This pattern aligns with the concept of ‘cultural humility’ in healthcare, prioritising ongoing self-reflection and individual assessment over fixed assumptions about cultural identity (Sarkin, 2019; Knight & Lucy, 2022; Borden & Dunn, 2024; Vatwani, 2024; Zhang, 2024). A study in the Netherlands found that cultural humility training for pharmacists improved their ability to provide patient-centred care and enhanced patient satisfaction among diverse populations (Van der Meer et al., 2023).

It is recommended that the UAE health authorities (MOHAP, SEHA, EHS) should mandate a certified cultural humility training module for pharmacists as part of continuing professional development (CPD) and license renewal, and should go beyond basic cultural competence, focusing on self-reflection, bias awareness, and respectful care for diverse groups such as migrant workers and linguistic minorities, and delivered through existing digital health platforms (e.g. Sheryan or TAMM) to ensure nationwide consistency and accountability.

Notably, pharmacists who had received formal cultural diversity training scored significantly higher in cultural awareness than those without, supporting prior evidence that structured education meaningfully enhances awareness and reflective capacity (Doroudgar et al., 2021; Drame, 2022). This is consistent with findings from a study in France, which found that cultural diversity training in both undergraduate studies and pharmacy school was associated with higher scores on the modified Clinical Cultural Competency Questionnaire (CCCQ), highlighting the importance of integrating cultural competence training into the pharmacy curriculum to enhance students’ overall cultural competence (Dubois et al., 2023). For example, a study found that cultural diversity training in both undergraduate studies and pharmacy school was associated with higher scores on the modified Clinical Cultural Competency Questionnaire (CCCQ), highlighting the importance of integrating cultural competence training into the pharmacy curriculum to enhance students’ overall cultural competence (Doroudgar et al., 2021).

The absence of statistically significant differences in culturally competent behaviour (CCB) scores across demographic and workplace variables, such as age, gender, nationality, English proficiency, work setting, or education level, suggests that cultural competence is not inherently tied to static personal or professional characteristics. This finding underscores the importance of intentional, ongoing efforts, such as workplace policies, mentorship, and inclusive communication practices, rather than relying on demographic diversity or assumed cultural exposure to foster competence (Kawathekar & Campbell, 2023). This is supported by a study in Brazil, which found that implementing policies that promote cultural competence, such as the Culturally and Linguistically Appropriate Services (CLAS) standards, can guide organisations in creating inclusive environments that respect and accommodate cultural diversity (Silva et al., 2022).

Implementing policies that promote cultural competence, such as the Culturally and Linguistically Appropriate Services (CLAS) standards, can guide organisations in creating inclusive environments that respect and accommodate cultural diversity.

A limitation of this study is its reliance on self-reported data, which may be influenced by social desirability bias (Lee & Woodliffe, 2010; Replication Data for: Threat-Inducing Violent Events Exacerbate Social Desirability Bias in Survey Responses., 2021; Zhu et al., 2024). Additionally, the cross-sectional design does not allow causality to be established (Savitz & Wellenius, 2023). This limitation is acknowledged in a study conducted in Italy, which also relied on self-reported data and highlighted the potential for social desirability bias in assessing cultural competence among healthcare providers (Rossi et al., 2022). The study recommended using mixed-methods approaches to triangulate findings and reduce bias.

Future research should employ longitudinal or intervention-based designs to evaluate the long-term effects of cultural competence training on patient outcomes. Qualitative studies could also provide deeper insights into how pharmacists navigate cultural challenges in practice. By addressing these gaps, future work can further support the development of equitable and patient-centred pharmacy services in multicultural healthcare systems. This is supported by a study in Spain, which used a longitudinal design to evaluate the impact of cultural competence training on patient outcomes and found significant improvements in patient satisfaction and health outcomes over time (García et al., 2023). The study recommended using longitudinal designs to assess the long-term effects of cultural competence training.

## Conclusion

This study presents the first comprehensive assessment of cultural competence among community pharmacists in the Emirates, revealing moderate to high levels of cultural awareness and behaviour. However, there is a significant training gap, with over 60% lacking formal education on cultural diversity. Notably, pharmacists who had received such training scored significantly higher in cultural awareness and self-perceived competence. In contrast, workplace support, such as adequate staffing and workflow, was also positively associated with competence levels. Demographic factors, such as age, gender, nationality, and international experience, showed no significant influence, underscoring that cultural competence is primarily shaped through structured training and supportive environments rather than personal background alone. As one of the first empirical studies in the UAE to quantify these relationships, our findings highlight the importance of incorporating cultural competence training into pharmacy curricula and workforce development strategies to enhance patient-centred care in an increasingly diverse healthcare landscape.

## Ethics approval and consent to participate

The study received ethical approval from the University of Sharjah Research Ethics Committee (Approval No. REC-21-03-03-01). All participants provided informed consent. Data collection was anonymous, and participation was voluntary.

## Acknowledgements

The authors sincerely thank the community pharmacists in Dubai, Sharjah, and Ajman for their valuable participation in this study. Their time, insights, and cooperation were essential to the success of this research. The authors also appreciate the support of Medical English Service for their professional proofreading of the manuscript.

## Author contributions

All authors meet the criteria for authorship as outlined by the ICMJE. Khalid Awad Al-Kubaisi and Abduelmula R. Abduelkarem led the study conception and design. Derar H. Abdel-Qader, Karem H. Alzoubi, and Nadia Al Mazrouei contributed to data collection, literature contextualisation, and methodology. Semira Abdi Beshir and Asim Ahmed Elnour contributed to data interpretation and drafting the manuscript. All authors critically reviewed, revised, and approved the final manuscript.

## Consent for publication: not applicable

This study does not include any individual data requiring consent for publication.

## Supplementary Material

Raw Data1.xlsx

## Data Availability

Supplementary materials, including the survey tool and statistical appendices, are available from the corresponding author upon reasonable request or as online supplementary files.
